# A computer-aided diagnosis of multiple sclerosis based on mfVEP recordings

**DOI:** 10.1371/journal.pone.0214662

**Published:** 2019-04-04

**Authors:** Luis de Santiago, E. M. Sánchez Morla, Miguel Ortiz, Elena López, Carlos Amo Usanos, M. C. Alonso-Rodríguez, R. Barea, Carlo Cavaliere-Ballesta, Alfredo Fernández, Luciano Boquete

**Affiliations:** 1 Grupo de Ingeniería Biomédica, Departamento de Electrónica, Universidad de Alcalá, Alcalá de Henares, Spain; 2 Instituto de Investigación Hospital 12 de Octubre (i+12), Madrid, Spain; 3 Facultad de Medicina, Universidad Complutense de Madrid, Madrid, Spain; 4 Departamento de Física y Matemáticas, Universidad de Alcalá, Alcalá de Henares, Spain; 5 RETICS: Red Temática de Investigación Cooperativa Sanitaria en Enfermedades Oculares Oftared, Madrid, Spain; University of Pécs Medical School, HUNGARY

## Abstract

**Introduction:**

The aim of this study is to develop a computer-aided diagnosis system to identify subjects at differing stages of development of multiple sclerosis (MS) using multifocal visual-evoked potentials (mfVEPs). Using an automatic classifier, diagnosis is performed first on the eyes and then on the subjects.

**Patients:**

MfVEP signals were obtained from patients with Radiologically Isolated Syndrome (RIS) (n = 30 eyes), patients with Clinically Isolated Syndrome (CIS) (n = 62 eyes), patients with definite MS (n = 56 eyes) and 22 control subjects (n = 44 eyes). The CIS and MS groups were divided into two subgroups: those with eyes affected by optic neuritis (ON) and those without (non-ON).

**Methods:**

For individual eye diagnosis, a feature vector was formed with information about the intensity, latency and singular values of the mfVEP signals. A flat multiclass classifier (FMC) and a hierarchical classifier (HC) were tested and both were implemented using the k-Nearest Neighbour (k-NN) algorithm. The output of the best eye classifier was used to classify the subjects. In the event of divergence, the eye with the best mfVEP recording was selected.

**Results:**

In the eye classifier, the HC performed better than the FMC (accuracy = 0.74 and extended Matthew Correlation Coefficient (MCC) = 0.68). In the subject classification, accuracy = 0.95 and MCC = 0.93, confirming that it may be a promising tool for MS diagnosis.

**Conclusion:**

In addition to amplitude (axonal loss) and latency (demyelination), it has shown that the singular values of the mfVEP signals provide discriminatory information that may be used to identify subjects with differing degrees of the disease.

## Introduction

### Multiple sclerosis and risk patients

Multiple sclerosis (MS) is a neurodegenerative disease characterized by chronic demyelination of the central nervous system (CNS) and which, as it develops, severely compromises patient quality of life. Although the cause of the disease remains unknown, it is assumed to be due to complex interactions between genetic and environmental factors. MS is not currently curable. The aim, therefore, is to diagnose it early and to provide treatment that reduces the risk of relapse and the progression of disability.

Radiologically isolated syndrome (RIS) defines individuals who do not show neurological symptoms but for whom magnetic resonance imaging (MRI) reveals findings that suggest future demyelination events. The conversion rate from RIS to MS was 65% after a mean follow-up of 5.3 years, and 88% after a mean follow-up of 14.1 years [1].

Clinically isolated syndrome (CIS) refers to a first episode of neurological symptoms that do not present fever and last a minimum of 24 hours. It is caused by inflammation or demyelination in the CNS. The most common symptoms are optic neuritis, a brainstem and/or cerebellar syndrome, a spinal cord syndrome or, occasionally, cerebral hemispheric dysfunction [2]. At median follow-up of 4.31 years, 623 out of 1047 CIS cases converted to MS [3]. Optic neuritis (ON) represents inflammation and demyelination of the optic nerve. In 38% of patients diagnosed with multiple sclerosis, ON is the first clinical manifestation of the disease [4].

### Multiple sclerosis diagnosis

The current criteria used to diagnose forms of MS were originally formulated by [5] and revised by [6] and [7]. Diagnosis should take into account evidence of damage to the CNS disseminated in time (on different dates) and in space (damage to at least two different parts of the CNS) and should exclude other conditions that, due to their clinical or laboratory profile, can mimic MS.

### Multifocal visual-evoked potentials (MfVEPs)

Multifocal visual-evoked potentials (mfVEPs) provide a method to diagnose optic pathway conditions. The visual stimulus is subdivided into a number (typically 60) of sectors. Each of these sectors is an independent stimulus controlled by specialized software. The electrical activity evoked by each stimulus in the visual cortex is recorded in the electroencephalograms (EEG). From a single, continuous EEG signal, a mathematical algorithm extracts the evoked response generated by each sector [8].

MfVEPs are a promising new tool of diagnostic and prognostic value in ON and MS [9]. MS leads to mfVEP abnormalities such as diminished intensity (amplitude), delayed nerve conduction velocity (latency) or morphological abnormalities and wave cancellation. MfVEP amplitude has been shown to be a functional biomarker of axonal loss in MS [10]. Latency is useful in assessing the extent of demyelination [11]. In [12], the authors demonstrated that mfVEP latency delay was evident in MS patients, where demyelination spreads along the entire visual pathway.

The various phases of record acquisition and processing have been enhanced to increase the effectiveness of diagnosis using mfVEPs. Previous papers have applied principal component analysis (PCA) to maximize sensitivity [13], Prony’s method to improve record quality [14], Gaussian wavelets to estimate latency reliably [15] and new types of stimuli to reduce test time [16]. Automated deep-learning image classification was used in [17] to analyse mfVEP plots; high AUC (area under the curve) and accuracy were achieved for assessing visual functions in patients with pituitary adenomas.

### Computer-aided diagnosis (CAD)

In MS, early treatment has been considered the best strategy [18], hence qualitative and faster clinical decisions are needed. In line with this, computer-aided diagnosis (CAD) has become one of the most important areas of research [19].

Several papers have implemented CAD systems to analyse MRI images to improve MS diagnosis. A false-positive reduction method for analysing MRI lesions was proposed in [20]. A new method for analysing MRI images based on the wavelet transform and PCA analysis, with logistic regression as classifier, was presented in [21]. In [22], the authors diagnose MS versus controls, comparing three machine-learning-based classifiers: the decision tree, k-NN and the support vector machine using the wavelet and wavelet entropy obtained with MRI. More recently, [23] classified MS subtypes based on features gathered from MRI and the Expanded Disability Status Scale using non-linear classification models (Convex Combination of Infinite Kernels).

Other papers analyse the EEG using entropy parameters to distinguish between control subjects and MS patients [24] or perform classification using a multilayer perceptron artificial neural net of retinal-nerve fibre layer data [25].

### Objective of this study

The objective of this study is to develop computer-aided diagnosis of multiple sclerosis based on mfVEP recordings. In the current study, a previously published patient database [10,26] is used, to which a new method of computer-assisted diagnosis is applied. The CAD system is implemented in two phases: in the first, the eyes are classified based on their mfVEP readings; in the second, diagnosis is performed on each subject.

Previous papers have demonstrated the existence of significant differences in the amplitude and latency parameters of mfVEP signals captured from control subjects and patients with differing levels of MS affectation [27]. MfVEP latency is potentially useful for assessing neuroprotective and remyelinating strategies in relapsing–remitting multiple sclerosis [28].

This paper uses the singular values of the mfVEP recordings, obtained using Singular Spectrum Analysis (SSA), as discriminating parameters in the eye classifier. The automatic classifier used is the k-Nearest Neighbour implemented on two separate structures: a flat multiclass classifier and a hierarchical classifier. Diagnosis is performed on each subject according to the results of classification of the subject’s eyes.

## Materials and methods

### Subjects database

The study protocol was approved by the Institutional Review Boards of University of Alcalá-affiliated hospitals and adhered to the tenets of the Declaration of Helsinki. The purpose and potential risks of the study were explained and all participants provided written informed consent. A cohort of eyes from patients with clinically definite MS (n = 56 eyes) and at different relative risk of developing MS, classified as RIS (n = 30 eyes) and CIS (n = 62 eyes), was included in this study and compared with a control group (n = 44 eyes) (Table 1). CIS and MS eyes were divided into two subgroups—ON eyes and non-ON eyes—based on whether or not they had had prior clinical ON episodes. ON episodes were always clinically defined by temporary reduced visual acuity in conjunction with other associated symptoms such as pain worsened by eye movement, variable visual field loss and colour perception changes.

**Table 1:** Subject demographics.

	Controls	RIS	CIS	MS
**Number of subjects**	22	15	31	28
**Male:female ratio**	9:13	5:10	11:20	7:21
**Age (years)**	30.30 (7.60)	39 (7.80)	30.29 (9.55)	24.39 (10.09)
**ON / non-ON eyes ratio**	0/44	0/30	13/49	37/19
**BCVA**	1.00 (0.0)	1.00 (0.0)	1.00 (0.0)	1.00 (0.0)
**EDSS**	--	--	2 (1.8)	0.7 (1.9)

Mean (standard deviation); ON: Optic Neuritis; BCVA: Best-Corrected Visual Acuity; EDSS: Expanded Disability Status Scale; RIS: Radiologically Isolated Syndrome; CIS: Clinically Isolated Syndrome; MS: Multiple Sclerosis.

All participants underwent an ophthalmologic examination that included best-corrected visual acuity (BCVA) using a high-contrast Snellen acuity chart. Inclusion criteria for RIS patients were based on [29]; MRI anomalies that did not account for clinically apparent impairments, and CNS white-matter anomalies with the following criteria: (1) ovoid, well-circumscribed and homogeneous foci with or without involvement of the corpus callosum; (2) T2-hypertensities measuring > 3 mm^2^ and fulfilling 3 of 4 Barkhof criteria [30] for dissemination in space; and (3) CNS anomalies not consistent with a vascular pattern. Eyes from CIS subjects having a first clinical episode suggestive of CNS demyelination involving the optic nerve, brainstem, spinal cord or other topography not attributable to other inflammatory diseases but lacking radiological evidence of dissemination of lesions over time were included in this study. Patients with CIS were included in the study within 3 months of their first clinical event. The MS groups comprised patients clinically diagnosed according to the McDonald criteria. MS patients had suffered one ON episode at least 6 months before being recruited. Full descriptions of the CIS and MS databases can be found in the following articles: CIS [26] and MS [31]. Finally, age-matched healthy subject eyes (n = 44 eyes) with normal neurological and ophthalmologic examination results were included as a control group.

### MfVEPs

The practical aspects of taking mfVEP recordings have been described in previous papers [8,32]. Briefly, VERIS software 5.9 (Electro-Diagnostic Imaging, Inc., Redwood City, CA) was used to obtain 6 channels for each of the 60 sectors into which the visual field is divided. A sampling frequency of 1200 Hz was used and 600 samples were obtained in each recording (length 500 ms). The signals were digital-passband-filtered (1–35 Hz) using a fast Fourier transform. MfVEPs were always recorded outside acute relapse ON.

### Obtaining the features of the mfVEP recordings

#### Amplitude

**Fig 1:**
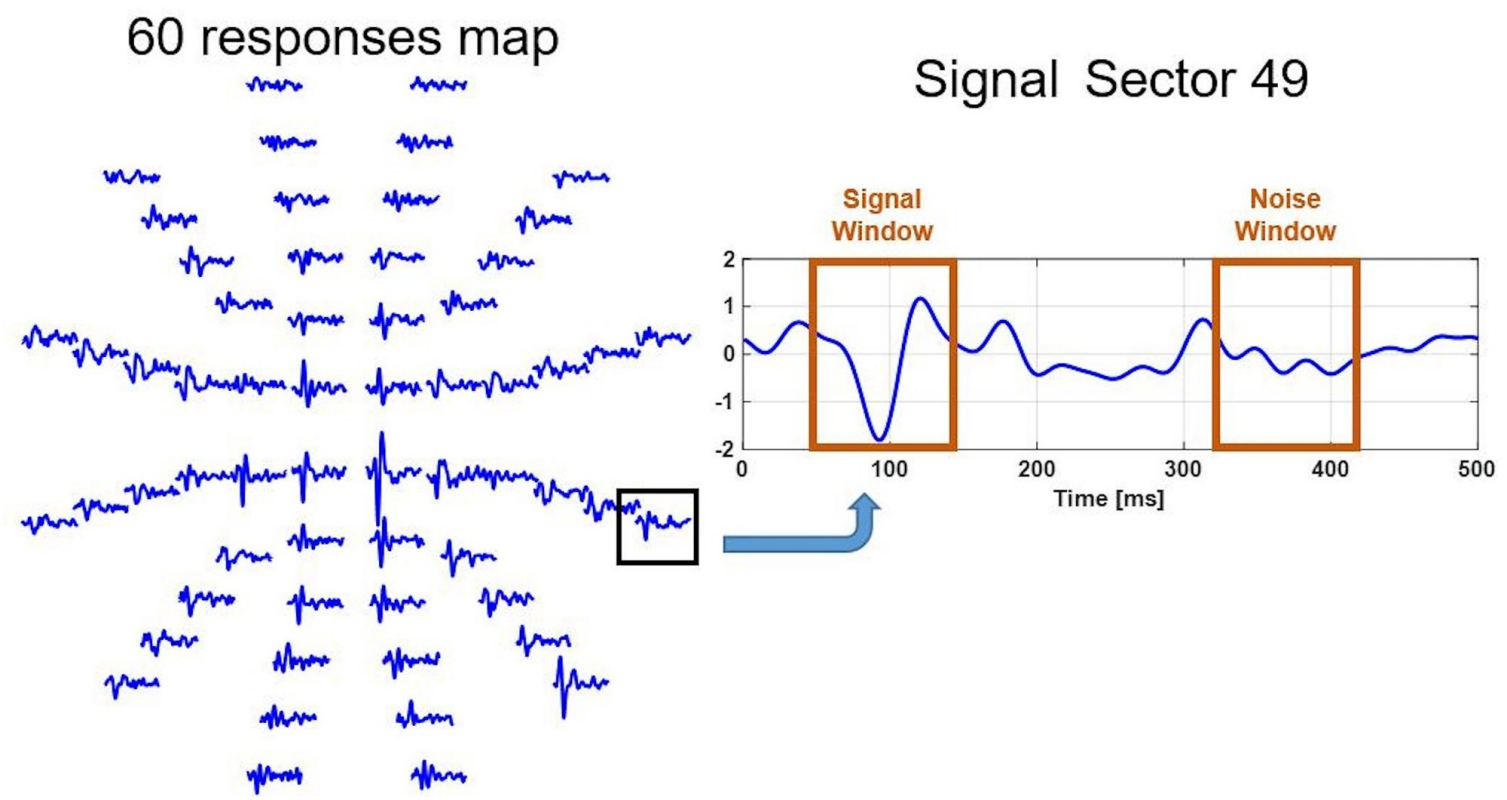
Best channel waveforms obtained from a control subject. Definition of the signal and the noise window.

The recording was divided into two different intervals: the signal window (45–150 ms), which contains the evoked potential response, and the noise window (325–430 ms), which essentially contains noise (Fig 1).

The amplitude is quantified as the signal-to-noise ratio (SNR) calculated as [33]:

$$\text{SNR}=\frac{\text{RMS}\;\left(X_{45-150\;\text{ms}}\right)}{\text{mean}\;\left(\text{RMS}\;\left(X_{325-430\;\text{ms}}\right)\right)}$$ (1)

where RMS(X_45–150 ms_) was the Root Mean Square (RMS) amplitude of the waveform in the signal window. The mean RMS(X_325–430 ms_) was the average RMS amplitude of all 60 waveforms in the noise windows.

The following calculations were performed using the channel with the highest SNR in each sector of the visual field (“the best channel”). If the SNR value of the best channel is under the threshold value of 1.7, the sector is considered a non-analysable sector (NAS) and it is discarded [33].

#### Latency

**Interocular latency** [ms] is computed using the cross-correlation method. This method shifts the response from one eye along the x axis to maximum overlap (best correlation) with the response from the other eye. Hence, the amount of shift represents the latency difference between the eyes [34]. The computed interocular latency value was assigned for both eyes (OD and OS). Absolute values were used to obtain averaged interocular values. The sign (indicating whether OD or OS is longer) was ignored (S1 File).

**Absolute monocular latency** [ms] is obtained as the relative monocular latency of responses. The cross-correlation was calculated between the subject’s response and a template [35]. The template was created for each location, eye and channel and was derived from averaging the responses of 100 control subjects [36].

### Singular spectrum analysis

**Fig 2:**
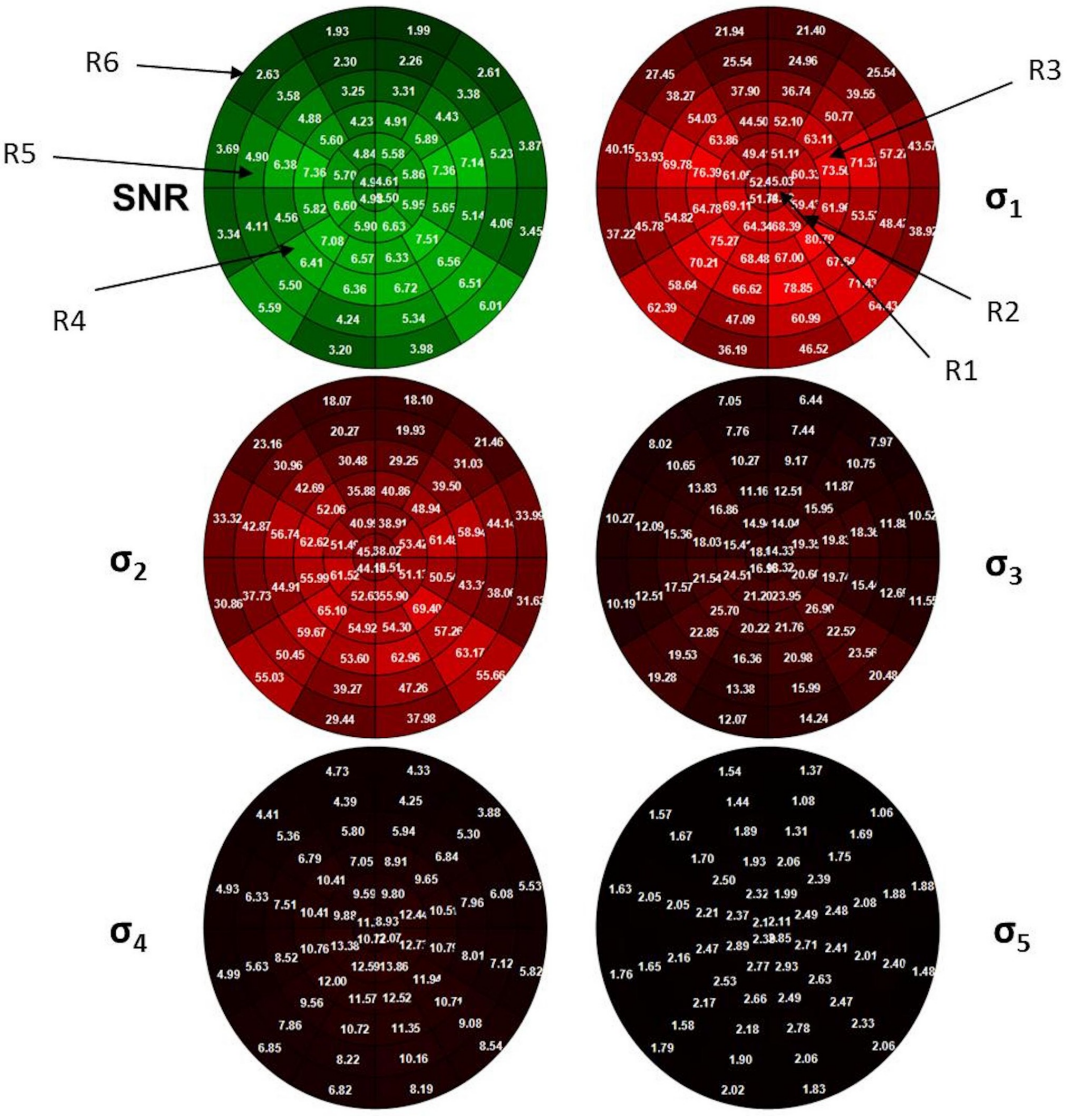
Signal-to-noise ratio (SNR) values (green) and singular values (σ_1_,…σ_5_) for the averaged control group for each sector of the visual field. When the values tend to zero, the colour of the sector is darker. R1,…R6: concentric rings.

Singular spectrum analysis facilitates time-series decomposition, trend detection, hidden component extraction, denoising and filtering. Several previous manuscripts present applications of SSA to analyse electrophysiological signals [37–40]. Singular values (σ_1_, σ_2_, σ_3_, …,σ_7_) obtained from SSA applied to mfVEP recordings were used to characterize the signal. Fig 2 shows the SNR values and the first 5 singular values (σ_1_, … σ_5_) in the 60 sectors of the visual field for the average of the entire control database. The singular values of a signal are approximately proportional to the mean value of a portion of the power spectrum. The lowest singular values (σ_1_, σ_2_ …) correspond to the lowest frequencies of the signal [41], so the first singular values contain trend information on the signal and the last singular values contain variance. The mathematical details of singular spectrum analysis are presented in S2 File.

### Feature vector

The feature vector of each eye was computed according to a topographical division. The visual field (Fig 2**)** was divided into six concentric rings of increasing retinal eccentricity from 1° (R1: the most central ring) to 22.2° (R6: the most peripheral ring).

Based on the parameters described, the following feature vector is obtained for each eye (Table 2).

**Table 2:** Feature vector.

**RING 1**			**RING 2**			**RING 3**			**RING 4**			**RING 5**			**RING 6**			**LABEL**
$P_{1}^{R1}$	…	$P_{i}^{R1}$	$P_{1}^{R2}$	…	$P_{i}^{R2}$	$P_{1}^{R3}$	…	$P_{i}^{R3}$	$P_{1}^{R4}$	…	$P_{i}^{R4}$	$P_{1}^{R5}$	…	$P_{i}^{R5}$	$P_{1}^{R6}$	…	$P_{i}^{R6}$	

Being:

$P_{1}^{RX}:$ Average SNR value in ring X (X = 1,…6).

$P_{2}^{RX}:$ Number of sectors in the ring whose SNR values are under the threshold limit (NAS sectors).

$P_{3}^{RX}:$ Average interocular latency value in ring X.

$P_{4}^{RX}:$ Average monocular latency value in ring X.

$P_{5}^{RX}:$ Average value of all first singular values (σ_1_) in ring X.

$P_{6}^{RX}$: Average value of all second singular values (σ_2_) in ring X.

… …

$P_{i}^{RX}:$ Average values of all i-th singular values (σ_i_) in ring X.

LABEL: Identifies the class (Controls, RIS, CIS-ON, CIS-non-ON, MS-ON, MS-non-ON) of the eye.

The number of singular values used in the feature vector varies from i = 1 to i = 7.

### Machine-learning eye classification

The k-NN algorithm is a distance- and example-based non-parametric method, proposed by Cover and Hart [42]. In a classification problem, there is an M number of **V** vectors with features whose class membership is known: {***V***_***j***_, *C*_*h*_}, *j* = 1,…*M*; *h* = 1, …, *H*. When presented with a new feature vector (***V***_***x***_), the objective of the classifier is to determine the class to which it belongs {*C*_*x*_ ∈ *C*_*h*_}. The distance between ***V***_***x***_ and the closest k vector ***V***_***j***_ is calculated and the output class membership assigned is the most frequent in this set of k neighbours. If k = 1, ***V***_***x***_ is assigned to the class of its nearest neighbour. The Matlab Classification Learner App (Mathworks Inc, Mass.) was used to train the model.

**Fig 3:**
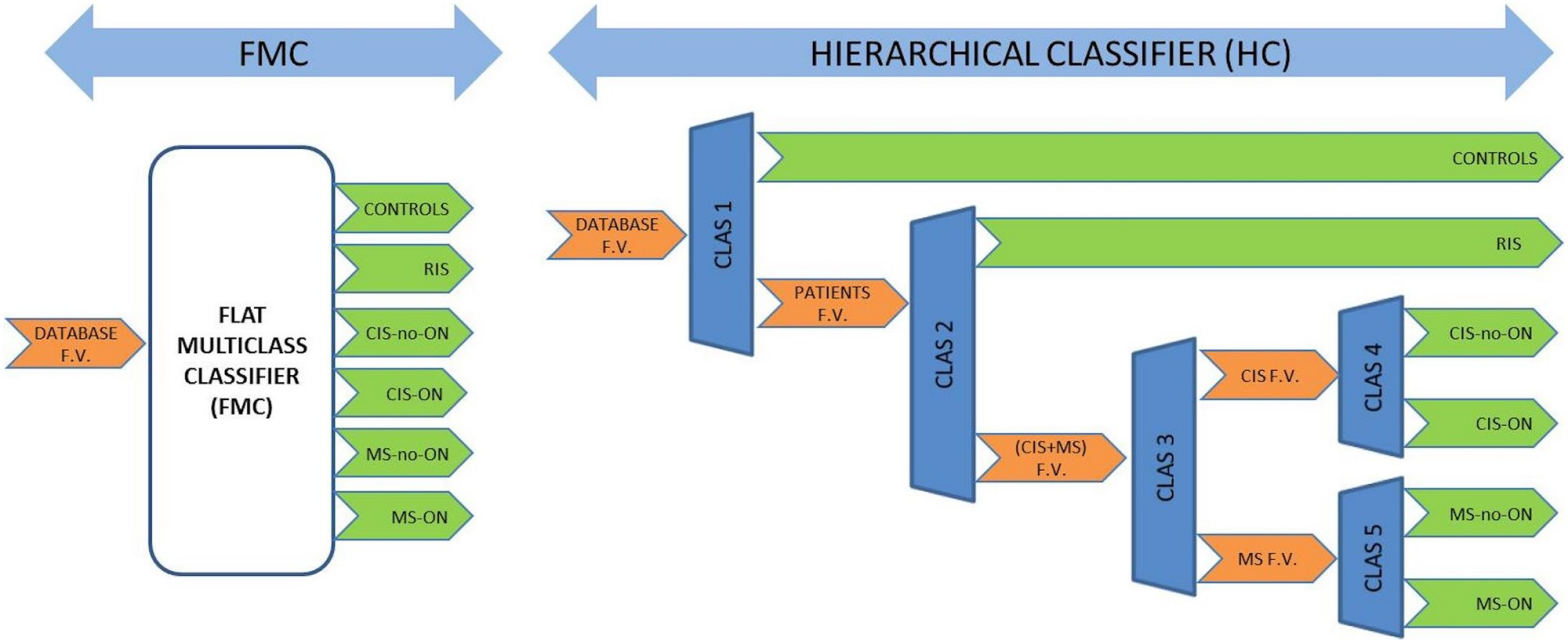
Flat multiclass classifier. (left) Hierarchical classifier (right). F.V.: Feature Vector; ON: Optic Neuritis; non-ON: non-Optic Neuritis; RIS: Radiologically Isolated Syndrome; CIS: Clinically Isolated Syndrome; MS: Multiple Sclerosis.

A flat multiclass classifier and a hierarchical classifier were tested (Fig 3).

The FMC input is the feature vector and the output comprises the 6 classes associated with differing degrees of development of MS into which the signals will be classified; implementation is achieved using a single k-NN algorithm.

The HC resolves the same overall classification problem in several classes using various appropriately ordered binary classifiers. It is implemented using 5 k-NN classifiers with varying k values and with different feature vectors.

The numbers of neighbours tested for each case are as follows: k = 1, k = 10 and k = all neighbours. The distance metric is Euclidean.

To assess classifier performance, cross-validation in *n* = 5 folds is used. Briefly, the cross-validation process is as follows: i) data are partitioned into *n* randomly chosen subsets (or folds) of roughly equal size, ii) one subset is used to validate the model trained using the remaining subsets. This process is repeated *n* times so that each subset is used exactly once for validation.

We use metrics that evaluate the classifier performance of each class (sensitivity, specificity and precision) and metrics that evaluate the overall performance: accuracy and Extended Matthew Correlation Coefficient (EMCC). EMCC is the generalization of the binary Matthew correlation coefficient to the multiclass case. It summarizes the confusion matrix. If the EMCC coefficient is equal to +1, classifier performance is perfect. If EMCC = -1, there is total disagreement between predictions and observations. The EMCC behaves consistently in practical cases and represents a good compromise between discriminancy, consistency and coherency with varying numbers of classes, unbalanced datasets and randomization [43].

### Subject classification

Diagnosis is performed on each subject according to the following criteria: if both eyes of the same subject receive the same classification, that classification is assigned to that subject; if the eye classifier assigns a different classification to each eye of the same subject, the diagnosis will be that of the eye with the highest SNR.

## Results

### Feature vector

Table 3 shows the values of the parameters (SNR, NAS, interocular and monocular latencies and singular values) used as the feature vector for the various databases.

**Table 3:** Mean (standard deviation) values of parameters for each database.

		Controls	RIS	CIS-non-ON	CIS-ON	MS-non-ON	MS-ON
**SNR**		5.00 (1.33)	4.34 (1.77)	3.82 (1.18)	2.63 (1.18)	3.51 (1.25)	3.18 (1.34)
**NAS**		3.00 (5.01)	10.10 (12.03)	10.37 (8.08)	25.23 (15.94)	12.11 (10.18)	15.51 (12.56)
**LAT**_**INTER**_ **[ms]**		4.13 (1.69)	7.08 (3.88)	9.97 (6.52)	15.85 (6.54)	11.76 (5.90)	11.49 (5.44)
**LAT**_**MONO**_ **[ms]**		-0.09 (4.94)	2.94 (5.23)	0.59 (4.58)	5.04 (6.07)	0.67 (5.59)	4.67 (7.29)
**σ**_**i**_	**σ1**	53.80 (18.57)	57.34 (23.70)	47.42 (17.93)	34.15 (16.29)	46.55 (24.30)	37.68 (17.63)
	**σ2**	44.41 (16.43)	47.00 (19.60)	38.95 (14.75)	27.72 (12.78)	39.43 (22.43)	31.00 (14.83)
	**σ3**	15.71 (5.94)	17.14 (6.96)	14.49 (5.76)	10.22 (3.83)	14.26 (8.18)	11.16 (5.83)
	**σ4**	8.54 (3.14)	10.01 (3.66)	8.42 (3.22)	6.90 (2.58)	7.74 (3.81)	6.67 (2.92)
	**σ5**	2.08 (0.74)	2.55 (0.93)	2.18 (0.88)	1.83 (0.73)	1.97 (0.76)	1.70 (0.62)
	**σ6**	0.55 (0.21)	0.74 (0.23)	0.61 (0.23)	0.58 (0.24)	0.53 (0.19)	0.49 (0.15)
	**σ7**	0.03 (0.01)	0.04 (0.01)	0.03 (0.01)	0.03 (0.01)	0.03 (0.01)	0.02 (0.01)

SNR: Signal to noise ratio; NAS: Number of Analysable Sectors; LAT_INTER: Interocular latency; LAT_MONO: Monocular latency; ON: Optic Neuritis; non-ON: non-Optic Neuritis; RIS: Radiologically Isolated Syndrome; CIS: Clinically Isolated Syndrome; MS: Multiple Sclerosis.

One-way analysis of variance (ANOVA) is used to compare the mean values of SNR, NAS, LAT_INTER_, LAT_MONO_, σ_1_,…, σ_7_ for the 6 different groups (CONTROL, RIS, CIS-non-ON, CIS-ON, MS-non-ON and MS-ON). The p-values obtained are less than 0.001 for all the parameters, except for σ_4_ (p = 0.0014) and σ_5_ (p = 0.0010); therefore, statistically significant differences between groups were found for each parameter, at the 0.05 level of significance.

**Fig 4:**
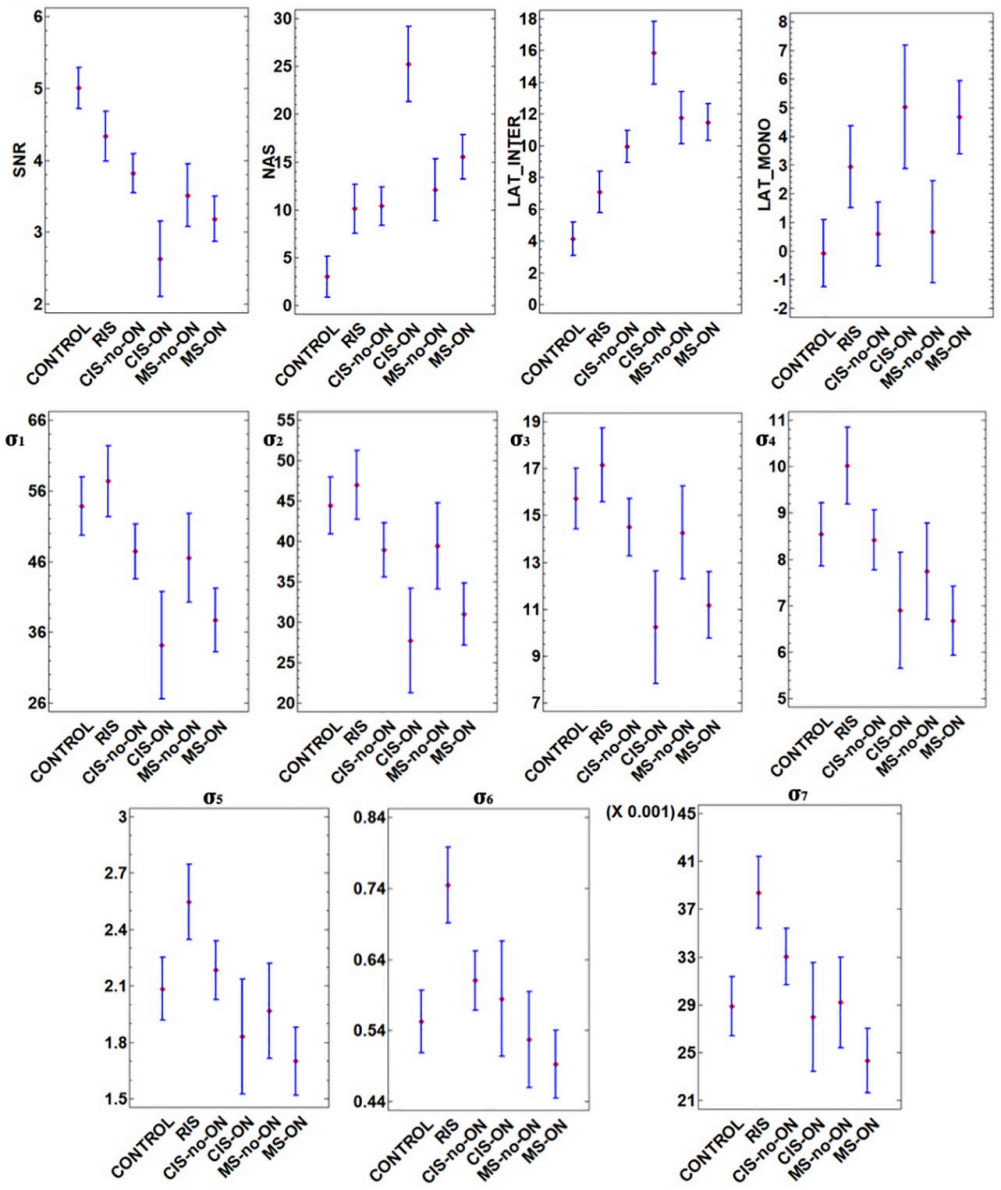
Means plot with 95% LSD intervals for the feature vector by group. SNR: Signal to Noise Ratio; NAS: Number of Analysable Sectors; LAT_INTER: Interocular latency; LAT_MONO: Monocular latency; ON: Optic Neuritis; non-ON: non-Optic Neuritis; RIS: Radiologically Isolated Syndrome; CIS: Clinically Isolated Syndrome; MS: Multiple Sclerosis.

Fig 4 shows the means plot with Least Significant Difference (LSD) intervals for each parameter by group, at the 95% confidence level. If two confidence intervals do not overlap, this offers evidence of a statistically significant difference between the means of the two groups, at the 0.05 level of significance.

SNR mean values decrease as risk of developing MS increases, with the lowest value being found in CIS-ON. Significant differences have been found in SNR values between controls and patients. No significant differences were observed between MS-ON and MS-non-ON eyes, because most non-ON eyes have been shown to be subclinically affected in clinically definitive MS groups [10]. Previous papers have found similar SNR values for MS patients with or without ON [44].

The lowest amplitude values were found in CIS groups. These signals were recorded within 3 months of the subject’s first clinical event, so the functional optic nerve fibres were still affected by axonal degeneration [45,46].

The lowest NAS mean value was found in the control subject group and increased as latency increased and amplitude decreased, with the highest value being found in the CIS-ON group. There is a significant difference between the NAS values of the control subjects and patients. A significant difference in NAS also exists between CIS-ON and the rest of the database.

In normal subjects, latencies in both interocular and monocular measurements should be essentially identical and close to 0 [47]. Low interocular values were obtained in the control group but were higher in patients. Significant differences were found between controls, RIS and CIS-ON. Interocular latency tended to be high in groups of patients with ON due to the functional differences between eyes in unilateral ON cases. Previous papers [48] found a significant increase in mfVEP latency in ON eyes compared to fellow (non-ON) eyes.

Monocular latencies were close to 0 in controls (-0.09 (4.94)) and non-ON eyes (CIS-non-ON: 0.59 (4.58) and MS-non-ON: 0.67 (5.59)). No significant differences were found between these 3 groups. High monocular latency values were found in ON-affected eyes (CIS-ON and MS-ON). A significant difference in monocular latency existed between patients with and without ON. This demonstrated that ON has a great impact on conduction velocity [10]. RIS patients presented moderate latency values and no significant difference with the other patient groups.

The singular values presented significant differences. There were no significant differences between the singular values of MS-non-ON and MS-ON eyes. **σ**_**1**_ **σ**_**2**_ **σ**_**3**_ performed similarly and discriminated between controls and RIS among ON patients (CIS-ON and MS-ON) and between CIS-non-ON and CIS-ON patients. **σ**_**4**_ **σ**_**5**_ **σ**_**6**_ **σ**_**7**_ likewise performed similarly and discriminated between the RIS group and the rest of the database.

### Eye classifier results

Tests were performed using various parameters (k values, differing singular values for the signal) with both the FMC and the HC. The best results for each classifier were obtained with the configurations presented in Table 4.

**Table 4:** Optimal configuration for eyes, by classifier.

Classifier		n-SV	K-NN	EYE CLASS PREDICTION
**Flat Multiclass Classifier**		**σ**_**1**_**,…, σ**_**4**_	1	All classes
**Hierarchical Classifier**	**CLAS 1**	**None** (i = 0)	1	Controls vs Patients
	**CLAS 2**	**σ**_**4**_**,…, σ**_**7**_	1	(CIS+MS) vs RIS
	**CLAS 3**	**None** (i = 0)	1	CIS vs MS
	**CLAS 4**	**σ**_**1**_**, σ**_**2**_**, σ**_**3**_	1	CIS-ON vs CIS-non-ON
	**CLAS 5**	**σ**_**1**_**, σ**_**2**_**, σ**_**3**_	10	MS-ON vs MS-non-ON

n-SV: number of Singular Values; k-NN: number of Nearest Neighbours; ON: Optic Neuritis; non-ON: non-Optic Neuritis; RIS: Radiologically Isolated Syndrome; CIS: Clinically Isolated Syndrome; MS: Multiple Sclerosis.

The best value for nearest number of neighbours is 1, except for CLAS 5 (HC), in which it is 10. A low K value means that the best prediction is made when “local” information is used. K = 1 provides the most flexible fit, which will have low bias but high variance. A higher K value averages more voters in each prediction and hence it is more resilient to outliers.

The optimal singular values used in the feature vectors vary between the different classifiers from 0 (CLAS 1, CLAS 3) to 4 (CLAS 2).

To visualize the performance of the classifiers, the confusion matrix for the FMC and the HC are presented in Table 5.

**Table 5:** Confusion matrix for each eye classifier.

CLASS		PREDICTED CLASS						
**FMC**	DATABASE	**Controls**	**RIS**	**CIS-ON**	**CIS -non-ON**	**MS-ON**	**MS -non-ON**	
	**Controls** **(n = 44)**	36	0	1	7	0	0	TRUE CLASS
	**RIS** **(n = 30)**	2	17	0	11	0	0	
	**CIS-ON** **(n = 13)**	1	0	1	9	2	0	
	**CIS-non-ON** **(n = 49)**	1	8	2	32	4	2	
	**MS-ON** **(n = 37)**	0	0	1	18	14	4	
	**MS-non-ON** **(n = 19)**	0	3	1	5	6	4	
**HC**		**Controls**	**RIS**	**CIS-ON**	**CIS -non-ON**	**MS-ON**	**MS -non-ON**	
	**Controls** **(n = 44)**	43	0	0	1	0	0	TRUE CLASS
	**RIS** **(n = 30)**	0	27	3	0	0	0	
	**CIS-ON** **(n = 13)**	0	0	6	7	0	0	
	**CIS-non-ON** **(n = 49)**	0	5	10	34	0	0	
	**MS-ON** **(n = 37)**	0	0	7	0	27	3	
	**MS-non-ON** **(n = 19)**	0	0	1	0	13	5	

ON: Optic Neuritis; non-ON: non-Optic Neuritis; RIS: Radiologically Isolated Syndrome; CIS: Clinically Isolated Syndrome; MS: Multiple Sclerosis; EMCC: Extended Matthew Correlation Coefficient; HC: Hierarchical Classifier; FMC: Flat Multiclass Classifier.

HC delivers more correct values than the FMC (the values of the main diagonal in the HC are higher than those in the FMC). The FMC produces a high error rate when classifying RIS as CIS-non-ON (11 cases) and MS-ON as CIS-non-ON patients (18 cases).

The highest error rate produced by the HC occurs when classifying cases of CIS-non-ON as CIS-ON (10 cases) and MS-non-ON as MS-ON (13 cases); these classifier errors in ON diagnosis can be rectified using other specific diagnostic tests for this disease.

The numerical values used to evaluate the classifiers’ performance are shown in Table 6.

**Table 6:** Evaluation of eye classifiers’ performance.

Parameter	Classifier	Controls	RIS	CIS-ON	CIS-non-ON	MS-ON	MS-non-ON	MEAN
**Sensitivity**	FMC	0.82	0.57	0.08	0.65	0.38	0.21	0.45
	HC	0.98	0.90	0.46	0.69	0.73	0.26	0.67
**Specificity**	FMC	0.97	0.93	0.97	0.65	0.92	0.97	0.90
	HC	1.00	0.97	0.88	0.94	0.92	0.98	0.95
**Precision**	FMC	0.90	0.61	0.17	0.39	0.54	0.40	0.50
	HC	1.00	0.84	0.22	0.81	0.68	0.63	0.70
**Accuracy**	FMC	0.54						--
	HC	0.74						--
**EMCC**	FMC	0.43						--
	HC	0.68						--

ON: Optic Neuritis; non-ON: non-Optic Neuritis; RIS: Radiologically Isolated Syndrome; CIS: Clinically Isolated Syndrome; MS: Multiple Sclerosis; EMCC: Extended Matthew Correlation Coefficient; HC: Hierarchical Classifier; FMC: Flat Multiclass Classifier.

The parameters that reflect the performance of the HC exceed those of the FMC. This becomes clear when the average parameter values per class are analysed: mean(Sensitivity_HC_) = 0.67 > mean(Sensitivity_FMC_) = 0.45; mean(Specificity_HC_) = 0.95 > mean(Specificity_FMC_) = 0.90; and mean(Precision_HC_) = 0.70 > mean(Precision_FMC_) = 0.50. The same is the case for the overall parameters: Accuracy_HC_ = 0.74 > Accuracy_FMC_ = 0.54 and EMCC_HC_ = 0.68 > EMCC_FMC_ = 0.43. The only exception to this general performance affects specificity when detecting CIS-ON (Specificity_HC_ = 0.88 < Specificity_FMC_ = 0.97) and MS-ON (Specificity_HC_ = Specificity_FMC_ = 0.92).

### Subject classifier results

Given that the best eye classification is obtained with the HC model, the output from that model is used to perform diagnosis on each subject. The confusion matrix for subject diagnosis is shown in Table 7.

**Table 7:** Confusion matrix for the subject classifier.

	PREDICTED CLASS				
	**Controls**	**RIS**	**CIS**	**MS**	
**Controls (n = 22)**	22	0	0	0	**TRUE** **CLASS**
**RIS (n = 15)**	0	14	1	0	
**CIS (n = 31)**	0	3	28	0	
**MS (n = 28)**	0	0	1	27	

RIS: Radiologically Isolated Syndrome; CIS: Clinically Isolated Syndrome; MS: Multiple Sclerosis.

For the controls in our database, classification is perfect: sensitivity_C_ = specificity_C_ = precision_C_ = 1. Of the 15 RIS-type subjects, one is classified as CIS, representing sensitivity_RIS_ = 0.93, specificity_RIS_ = 0.96 and precision_RIS_ = 0.82. In the CIS patient group (n = 28), 3 are classified as RIS-type subjects, representing sensitivity_CIS_ = 0.90, specificity_CIS_ = 0.97 and precision_CIS_ = 0.93. Finally, the results of MS patient classification are as follows: sensitivity_MS_ = 0.96, specificity_MS_ = 1.0 and precision_MS_ = 1.0. The overall parameters that reflect the characteristics of the subject classification are MCC = 0.93 and accuracy = 0.95.

## Discussion

Although many biomarkers have been proposed for MS, at present only oligoclonal bands, magnetic resonance imaging, optical coherent tomography (OCT) and the JCv antibody index have been implemented in clinical practice [49]. MRI is the clinical test most widely used in diagnosing and monitoring MS [50], even though measures of white matter lesions do not correlate strongly with patients’ clinical symptoms (the MRI paradox [51]) and so ambiguous cases are frequently found in clinical practice [52]. For example, in [53] a modest correlation (r = -0.30) is obtained between MRI measurements of total brain white-matter lesions and cognitive function in MS patients.

Evoked potentials are considered to obtain better correlation values with the Expanded Disability Status Scale than structural data [54]. In addition, the most recent review of the McDonald criteria [7] recommends conducting studies of visual-evoked potentials in support of MS diagnosis. In line with this, the objective of this paper has been to advance implementation of a computer-aided system for diagnosing multiple sclerosis using mfVEP features and automatic classifiers that may help to address this clinical need.

Typical parameters used to study and quantify mfVEP recordings are signal amplitudes (related to axonal loss processes) and latencies (due to demyelination). These parameters show some capacity to discriminate between patient groups and identify significant differences in some cases when comparisons are made group by group. To the best of our knowledge, there have not been any prior studies into the relationship between the singular values of the mfVEP signals and the underlying electrophysiological processes. It has been shown that the singular values of the mfVEP signals provide discriminatory information that may be used to identify subjects with differing degrees of the disease.

This set of parameters (amplitude, latency and singular values) was used as input for a system that successfully discriminated between groups of eyes at different degrees of risk of MS.

Two different approaches were tested to implement the automatic eye classifier: FMC and HC. Parameters that reflect the performance of the HC exceed those of the FMC. This improvement may be because it adopts a divide-and-conquer approach to the problem by splitting the overall problem into smaller sub-problems. It also allows customization of various parameters of the intermediate classifiers: number of neighbours (K) and number of singular values used in the feature vector.

The relatively high EMCC value in HC (EMCC = 0.68) indicates that this classifier performs well. The specificity (0.95 mean value) is good for all the cases and higher than the sensitivity (0.67 mean value). This means that this classifier is better at detecting negative cases than at detecting positive ones.

Diagnosis of each subject is based on classification of both eyes. The overall parameters that characterize the confusion matrix (Table 7) are MCC = 0.93 and accuracy = 0.95.

Several previous papers have employed machine learning solutions to perform classification between controls and MS patients. A pattern recognition technique is used in [55] to learn a discriminant function that obtains a sensitivity of 0.82 and a specificity of 0.86 to distinguish between MS patients and controls using functional MRI (fMRI). In [56], SVMs are used as classifiers with diffusion tensor imaging (DTI) and fMRI data input between multiple sclerosis (RRMS) patients, obtaining accuracy = 89% ± 2%. In [25], measurements of the retinal nerve fibre layer (RNFL) are obtained. These are classified using a neural network to obtain AUC = 0.945 in the MS patients as compared with healthy subjects. Bayesian statistics-based biomarker creation was used in [57] to diagnose classes of either MS patients or controls according to alterations in bioactive lipid metabolism, achieving a sensitivity, specificity and accuracy of approximately 95% in training and test datasets. Recently, Ahmadi et al. [58] evaluated the phase–amplitude coupling of EEG signals in MS patients and controls; using an extreme learning machine neural network with online learning, they obtain accuracy = 0.912.

In this paper, classification between controls and patients is perfect: sensitivity_C_ = specificity_C_ = precision_C_ = 1. It is difficult to determine whether the advantage obtained over previous studies is due to the type of test (MRI, electrophysiology), to the type of machine learning applied or to the heterogeneity of the databases used.

Our study also classifies patients into 3 types: RIS, CIS and patients diagnosed with definitive MS. Of the RIS-type subjects, one is classified as CIS-type, representing sensitivity_RIS_ = 0.93, specificity_RIS_ = 0.96 and precision_RIS_ = 0.82.

For the CIS patient group (n = 28), 3 are classified as RIS-type subjects, representing sensitivity_CIS_ = 0.90, specificity_CIS_ = 0.97 and precision_CIS_ = 0.93. The 3 misclassified subjects are CIS subjects who have not suffered optic neuritis and whose potentials are largely unaltered.

Finally the results of classification of the MS patients are sensitivity_MS_ = 0.96, specificity_MS_ = 1.0 and precision_MS_ = 1.0.

To the best of our knowledge, very few papers have applied machine learning to classification of patients with differing levels of the disease. In [56], SVMs are used as classifiers based on diffusion tensor imaging (DTI) and fMRI data. Weak identification accuracies are obtained (63% ± 5%) when comparing MS patients with different levels of EDSS. Barbour et al. [59] develop diagnostic molecular-based (cerebrospinal fluid) classifiers. The classifier obtains the following AUC values for each possible classification: RRMS vs progressive MS: AUC = 0.91; MS vs CNS diseases that mimic MS: AUC = 0.98; relapsing–remitting vs MS: AUC = 0.91 and primary progressive vs secondary progressive MS: AUC = 0.5.

The main advantages of our method are that a) it obtains good values in patient diagnosis (Table 7), which evidently need to be confirmed by other clinical trials, and b) it is a fully automatic non-invasive method that does not require human intervention, as calculation of the elements of the characteristics vector and subsequent diagnosis is automatic. This avoids the need for tedious signal analysis by practitioners.

All these results show a promising machine-learning approach to identifying multiple sclerosis patients with a high degree of accuracy. Moreover, since the combined diagnostic technique (MRI, visual-evoked potentials, OCT) produces a huge amount of data it is beneficial to have access to machine-learning solutions that support practitioners’ decision-making.

### Limitations and future work

RIS patients present higher singular values than controls in all the cases (σ_1_,σ_2_…, σ_7_). To the best of our knowledge, this was the first time that SVD was applied to mfVEP signals, so it was not possible to compare these results with other papers. In our opinion, these SVD values were not related to the presence of noise or artefacts because the SNR values were high (4.34 ± 1.77) and mfVEP signals are only considered artefacted or noisy if the SNR is below 1.7 [60]. Previous applications of SVD to other electrophysiological signals did not state this fact [41,61]. At this stage of the study, there is no clear hypothesis about the physical meaning of this result and future work must investigate these findings.

One possible improvement to the HC would be to add more classifiers, particularly to classifiers with low sensitivity and high specificity. For example, in classifier 5, eyes classified as MS-ON (Sensitivity = 0.28 and Specificity = 0.9) would be input into an additional classifier to increase accuracy. These additional classifiers could be based on other parameters (wavelet coefficients, entropy values).

This paper has only used the basic family of K-NN classifiers. Testing other types of complex classifier, such as neural networks or support vector machines, could improve the accuracy of the results.

The database used is small and taken from a single centre. To avoid bias deriving from the differences between databases, the database should be extended to include subjects from other centres and signals recorded using other commercially available equipment.

This new independent database will show the real diagnostic utility of the analysis method presented.

Due to the small size of the database, it has been considered advantageous to collect data from both eyes. This increases the number of cases in the study, but may decrease its statistical power [62] when classifying eyes. This is a limitation of this study that will be addressed in future work.

Perhaps the best enhancement, considering the scientific literature available, would be to use a different series of clinical data and MRI recordings justified by the results used in clinical settings to diagnose MS and by the publications referred to in the state of the art.

## Conclusions

The objective of this paper has been to advance implementation of a computer-aided system for diagnosing multiple sclerosis using mfVEP features and automatic classifiers. In addition to amplitude (axonal loss) and latency (demyelination), it has shown that the singular values of the mfVEP signals provide discriminatory information. The best results for eye classification were obtained using a combination of K-NN classifiers and a hierarchical structure. In a second stage, a subject classifier based on the previous eye classification was implemented. The good results obtained with the subject classifier shows a promising machine-learning approach to diagnosing multiple sclerosis with a high degree of accuracy. This type of system can also be used to evaluate medical treatment response and can be employed in clinical trials.

## Supporting information

S1 File Appendix 1. Interocular latency computing notes.
(DOCX)

S2 File Appendix 2. Singular spectrum analysis.
(DOCX)
