# Supplementary material for: A computer-aided diagnosis of multiple sclerosis based on mfVEP recordings
Source: PLoS One. 2019 Apr 4;14(4):e0214662. doi: 10.1371/journal.pone.0214662 (PMC6449069; doi:10.1371/journal.pone.0214662)
Supplement: S1 File — Interocular latency computing notes. (DOCX) [file pone.0214662.s001.docx]

Supporting Information 1

Appendix 1:

Interocular latency computing notes.

The sign (+/-) in interocular latency measurements is just the information about which eye is delayed. However, this could be a problem when the data are averaged. This is explained with an example comprising two imaginary patients with acute unilateral ON. In patient number 1, the OD (ON affected) is delayed by 7 ms with respect to OS (latency = -7 ms). In patient number 2, the OS (ON affected) is delayed by 7 ms with respect to OD (latency = 7 ms). If we average latency, the result is 0 ms (no clinical information). Instead, if unsigned latency values are averaged, the result is 7 ms (clinical information is preserved). Since the unsigned values are only used for interocular latencies, higher latencies are obtained than in monocular cases.
