# Supplementary material for: A computer-aided diagnosis of multiple sclerosis based on mfVEP recordings
Source: PLoS One. 2019 Apr 4;14(4):e0214662. doi: 10.1371/journal.pone.0214662 (PMC6449069; doi:10.1371/journal.pone.0214662)
Supplement: S2 File — Singular spectrum analysis. (DOCX) [file pone.0214662.s002.docx]

Supporting Information 2

Appendix 2:

Singular spectrum analysis.

Singular spectrum analysis (SSA) facilitates time-series decomposition, trend detection, hidden component extraction, denoising and filtering.

SSA of biomedical signals has been used to analyse electroencephalograms to discriminate between subjects with normal and abnormal sleep condition [1]. In [2] the authors propose an adaptive SSA method to remove artefacts and extract rhythms from EEG signals. In [3] SSA is applied to smooth EEG. Meanwhile, [4] used SSA to filter and obtain the features of auditory-evoked potentials to diagnose hyperthyroidism.

SSA is computed in two steps: an embedding operation followed by singular value decomposition (SVD). In this paper, SSA is applied only in the signal window (where the evoked response is expected).

In the embedding stage, one-dimensional mfVEP series X(k) of length N = 128 samples is mapped into a Hankel trajectory matrix (**C**):

| $\mathbf{C=}\left( \boldsymbol{X}_{\boldsymbol{1}}\mathbf{,}\boldsymbol{X}_{\boldsymbol{2,}}\boldsymbol{\ldots,}\boldsymbol{X}_{\boldsymbol{T}} \right)=\left( \begin{matrix} x\left[ 1 \right] & x\left[ 2 \right] & \cdots& x\left[ T-1 \right] & x\left[ T \right] \\ x\left[ 2 \right] & x\left[ 3 \right] & \cdots& x\left[ T \right] & x\left[ T+1 \right] \\ \vdots& \vdots& \ddots& \vdots& \vdots\\ x\left[ L \right] & x\left[ L+1 \right] & \cdots& x\left[ N-1 \right] & x\left[ N \right] \end{matrix} \right)_{\left( L \right)x(T)}$ | (1) |
| --- | --- |

Matrix **C** is symmetrical and constant across the anti-diagonals. Columns **X**_i_ are called lagged vectors and contain L components (window length or embedding dimension). Thus, T = N – L + 1. The window length value is selected based on the frequency of interest (L ≥ fs /F_HIGH_; fs: sampling frequency; F_HIGH_: highest frequency of the original signal) to capture at least one period of the expected component [5]. In this paper, the values selected were: L = 64 and K = 65. The choice of window length is important, but the result is stable with respect to small changes of L [6].

The second step is to apply the SVD to the trajectory matrix. Matrix $\mathbf{C}_{\left( L \right)x(T)}$ can be decomposed into the product of three other matrices:

$$\mathbf{C}=\boldsymbol{U\Sigma}\mathbf{V}^{\mathbf{T}} (2)$$

Matrices $\mathbf{U}_{\left( L \right)x\left( L \right)}$(left singular vector matrix) and $\mathbf{V}_{(T)x(T)}$ (right singular vector matrix) are unitary ($\mathbf{U}^{H}\mathbf{U}=\mathbf{U}\mathbf{U}^{H}=\mathbf{I}_{L}, \mathbf{V}^{H}\mathbf{V}=\mathbf{V}\mathbf{V}^{H}=\mathbf{I}_{T}$) and $\boldsymbol{\Sigma}_{\left( L \right)x(T)}=diag\left( \sigma_{1}, \sigma_{2},\ldots\sigma_{\min\left\{ L, \left( T \right) \right\}}) \right),$the singular values of trajectory matrix **C** being arranged in descending order: $\sigma_{1} \geq\sigma_{2} \geq\sigma_{3}\ldots\geq0$. These singular values are used to characterize the signal.

**References:**

1. Kouchaki S, Sanei S, Arbon EL, Dijk D-J. Tensor Based Singular Spectrum Analysis for Automatic Scoring of Sleep EEG. IEEE Trans Neural Syst Rehabil Eng. 2015;23: 1–9. doi:10.1109/TNSRE.2014.2329557

2. Hu H, Guo S, Liu R, Wang P. An adaptive singular spectrum analysis method for extracting brain rhythms of electroencephalography. PeerJ. 2017;5: e3474. doi:10.7717/peerj.3474

3. Rasoulzadeh V, Erkus EC, Yogurt TA, Ulusoy I, Zergero SA. A comparative stationarity analysis of EEG signals. Ann Oper Res. 2017;258: 133–157. doi:10.1007/s10479-016-2187-3

4. Güven A, Altınkaynak M, Dolu N, Ünlühızarcı K. Advanced Analysis of Auditory Evoked Potentials in Hyperthyroid Patients: The Effect of Filtering. J Med Syst. 2015;39: 13. doi:10.1007/s10916-014-0184-0

5. James CJ, Lowe D. Extracting multisource brain activity from a single electromagnetic channel. Artif Intell Med. 2003;28: 89–104. doi:10.1016/S0933-3657(03)00037-X

6. Golyandina N, Korobeynikov A. Basic Singular Spectrum Analysis and forecasting with R. Comput Stat Data Anal. 2014;71: 934–954. doi:10.1016/j.csda.2013.04.009
